# Species-level characterization of the core microbiome in healthy dogs using full-length 16S rRNA gene sequencing

**DOI:** 10.3389/fvets.2024.1405470

**Published:** 2024-09-02

**Authors:** Connie A. Rojas, Brian Park, Elisa Scarsella, Guillaume Jospin, Zhandra Entrolezo, Jessica K. Jarett, Alex Martin, Holly H. Ganz

**Affiliations:** AnimalBiome, Oakland, CA, United States

**Keywords:** canine fecal microbiome, core microbiome, dogs, PacBio, 16S rRNA gene sequencing, diet, geography, body weight

## Abstract

Despite considerable interest and research in the canine fecal microbiome, our understanding of its species-level composition remains incomplete, as the majority of studies have only provided genus-level resolution. Here, we used full-length 16S rRNA gene sequencing to characterize the fecal microbiomes of 286 presumed healthy dogs living in homes in North America who are devoid of clinical signs, physical conditions, medication use, and behavioral problems. We identified the bacterial species comprising the core microbiome and investigated whether a dog’s sex & neuter status, age, body weight, diet, and geographic region predicted microbiome variation. Our analysis revealed that 23 bacterial species comprised the core microbiome, among them *Collinsella intestinalis*, *Megamonas funiformis*, *Peptacetobacter hiranonis*, *Prevotella copri*, and *Turicibacter sanguinis*. The 23 taxa comprised 75% of the microbiome on average. Sterilized females, dogs of intermediate body sizes, and those exclusively fed kibble tended to harbor the most core taxa. Host diet category, geographic region, and body weight predicted microbiome beta-diversity, but the effect sizes were modest. Specifically, the fecal microbiomes of dogs fed kibble were enriched in several core taxa, including *C. intestinalis*, *P. copri*, and *Holdemanella biformis*, compared to those fed raw or cooked food. Conversely, dogs on a raw food diet exhibited higher abundances of *Bacteroides vulgatus*, *Caballeronia sordicola*, and *Enterococcus faecium*, among others. In summary, our study provides novel insights into the species-level composition and drivers of the fecal microbiome in healthy dogs living in homes; however, extrapolation of our findings to different dog populations will require further study.

## Introduction

Research on the gut microbiome, particularly over the past 20 years has led to the recognition that bacteria and other microbes inhabiting the gastrointestinal tract are not just passive travelers and instead interact with the host in ways that can have a profound effect on health (1). These microbial communities in animals are complex, characteristic, and reflect the host’s diet, phylogenetic history, and other ecological factors (2–4). Of particular interest to human and animal health are studies examining whether there is a core microbiome that comprises essential groups of bacteria found in most healthy individuals (5), that may be functionally important to the host. There is some evidence to suggest that functional redundancy is common in microbial communities, and entire groups of bacteria may have overlapping functions such that in some cases one or more groups can stand in for another (6). However, in certain cases, there may be less redundancy in functions that are more specialized within these microbial communities. For example, it has been observed that *Peptacetobacter hiranonis* (formerly *Clostridium hiranonis*) is the primary or perhaps only bacterial species performing bile acid metabolism in domestic dogs (7, 8) and its elimination or depletion resulting from antibiotic use leaves the gut microbiome with reduced ability to perform this function (9).

A challenge for assessing the impact of disease, medications, and probiotics on the gut microbiome arises from a lack of a consensus on the definition of what comprises a “normal” or “healthy” core microbiome (10). Microbiome studies differ in the prevalence and abundance thresholds used to determine the core, as well as the taxonomic unit at which the core is being defined (e.g., at level of genera, species, or family) (5). Others may forgo taxonomy altogether and focus on core functional genes (5). Additionally, the criteria used to define a “healthy” from a “not healthy” individual are also contentious. The size, homogeneity, and descriptive characteristics of the host group may also influence the study’s findings. However, despite these challenges, efforts made to characterize the core microbiome of a diverse study population can be insightful and expand our understanding of the microbiome in that host species.

In this study, we screened the microbiomes of over 3,000 pet dogs (*Canis lupus familiaris*) living in homes in North America and focused on a curated subset of this group (*n* = 286) to provide a taxonomically and statistically defined core microbiome associated with health. Previous research has established the importance of the gut microbiome in canine health, yet significant gaps remain in identifying the precise bacterial species that constitute a healthy canine gut microbiome. Many past studies have relied on short-read sequencing technologies, which offer limited taxonomic resolution. Here, we employed full-length 16S rRNA gene sequencing (V1-V9) using PacBio technology to overcome these limitations and gain species-level insights. Long-read sequencing, particularly PacBio technology, has been shown to enhance the resolution and accuracy of microbiome analyses, deepening our understanding of the microbial communities present (11). Despite these advantages, the application of full-length 16S rRNA gene sequencing in dogs remains unexplored. Additionally, while factors like diet, body condition, probiotics, and antibiotics have already been studied for their impact on microbiome variation, their effects using full-length sequencing technology have not yet been examined.

Our curated subset of dogs were reported to be healthy by their owners (*n* = 230) or via veterinarian records (*n* = 56), and met strict criteria that included having no physical conditions and clinical signs, and no usage of daily medications or antibiotics, given that these contribute to gut dysbiosis in healthy dogs (12). With this dataset, we investigated whether factors such as sex & neuter status, age, body weight, diet, and geographic region significantly predicted microbiome alpha- or beta-diversity. In addition, although probiotic use is common in healthy dogs and dogs with chronic enteropathy (13), we aimed here to understand the bacterial composition of the fecal microbiome that is reflective of a less managed state. Thus, our healthy reference set excluded dogs that consumed probiotics. However, we conducted additional analyses to compare the fecal microbiomes of dogs in this reference set with those of dogs given bacterial probiotics (*n* = 86) who would have otherwise met the criteria for inclusion in the reference set. Collectively, our comparisons sheds light on the effects of important factors on the canine gut microbiome, providing valuable insights for the field of veterinary medicine.

## Methods

### Sample and metadata collection

Fecal samples were collected from 3,754 dogs, although samples from only 286 dogs (7.61% of samples) were the focus of this study (see section below). Of these 286 dogs, 230 were owned by customers of AnimalBiome, a private company offering microbiome testing services for companion animals, and 56 were enrolled in AnimalBiome’s stool bank program, which provides screened fecal material for veterinary purposes. Pet owners were instructed to collect a small amount of their dog’s fecal sample and place it in a 2 mL screw cap tube containing 70% molecular-grade ethanol. Pet parents were instructed to use the provided clean gloves, wooden sticks, and bags for fecal collection to avoid contamination.

Pet owners then shipped the fecal samples to AnimalBiome’s facilities in Oakland, CA and provided pertinent information about their dogs, including name, date of birth, body weight, body condition, spay or neuter status, breed, diet, medication and supplement use, current clinical signs, health diagnoses, and physical conditions via an online survey. Owners also documented the fecal consistency and color of the submitted samples.

### Criteria for defining the healthy dataset

For a dog to qualify for the healthy reference set, the following strict criteria needed to be met: body condition scores 4–6 (inclusive), no antibiotics given within the previous 12 months, no other medications reported currently in use, no bacterial probiotics, no AnimalBiome Gut Restore supplements, no clinical signs, no physical conditions, and no fecal descriptions that included the word “blood.” An additional 86 dogs met the aforementioned criteria with the exception of bacterial probiotics, and their microbiomes were compared to those of the healthy reference set to assess the impact of probiotics on the microbiome. For descriptive statistics regarding our reference set (see Table 1).

**Table 1:** Characteristics of the pet dogs that comprised the healthy reference set (*n* = 286).

Characteristic	Subcategory	*N* (%)
Age, in years*	Median & (range)	4 (0.6–11)
Body condition score	Median & (range)	5 (4–6)
Body weight (kg)*	Median & (range)	21.2 (1.8–67.1)
Body weight category	<6 kg	30 (10%)
	6–10 kg	33 (12%)
	10–25 kg	114 (40%)
	25–45 kg	99 (34%)
	45 kg +	10 (4%)
Sex & Neuter Status*	Female intact	27 (9%)
	Female sterilized	102 (36%)
	Male intact	40 (14%)
	Male sterilized	117 (41%)
Breed (broad)	Shepherd	33 (12%)
	Poodle (& mixes)	30 (10%)
	Retriever	28 (10%)
	Terrier	23 (8%)
	Mix (unknown breeds)	19 (7%)
	Other (Husky, PitBull, Collie, Bulldog, etc.)	153 (53%)
Diet*	Kibble only	85 (30%)
	Raw food only	76 (27%)
	Cooked food only	45 (16%)
	Other (combinations of kibble, raw, cooked)	80 (27%)
Geographic region*	USA West	111 (39%)
	USA MidWest	22 (8%)
	USA South	28 (10%)
	USA Northeast	37 (13%)
	Canada	7 (2%)
	North America (unknown location)	81 (28%)

Fecal samples from two-hundred and eighty-six dogs comprised the healthy reference set and had their microbiomes analyzed in this study. The dogs were privately owned and resided in North America.

An * indicates that this factor was included in statistical models that analyzed microbiome composition, alpha-diversity, and beta-diversity.

The 56 dogs that were part of AnimalBiome’s stool donor program met additional criteria and were clinically verified to be healthy via medical history records, veterinary visits, and monthly screenings of parasites and pathogens.

### DNA extraction and full-length 16S rRNA amplicon gene sequencing

Genomic DNA was extracted from canine fecal samples using the QIAGEN DNeasy Powersoil Pro Isolation Kit on the QIAcube HT instrument (QIAGEN, CA, USA). Amplification of the full-length 16S rRNA gene was achieved using primers 27F (5′-AGRGTTYGATYMTGGCTCAG-3′) and 1492R (5′-RGYTACCTTGTTACGACTT-3′), which were tailed with 16-bp asymmetric barcode sequences. The PCR mixture comprised 12.5 μL of KAPA HiFi HotStart ReadyMix PCR (KAPA Biosystems, MA, USA), 3 μL of each primer (at 2.5 μM), 2 μL of template DNA, and 4.5 μL of PCR-grade water. PCR conditions included an initial denaturation at 95°C for 3 min, followed by 25 cycles of denaturation at 95°C for 30 s, annealing at 57°C for 30 s, and a final extension at 72°C for 60 s. Full-length 16S rRNA purified amplicons were sequenced on a PacBio Sequel IIe platform (Pacific Biosciences, CA, USA). The sequenced amplicons were compared against the ZymoBIOMICS Microbial Community DNA Standard positive control (Zymo Research, Irvine, CA) and a negative control (PCR-grade water) to ensure QC and no contamination.

Although samples spanned multiple sequencing runs, this did not affect microbiome composition (PERMANOVA Bray-Curtis *F* = 0.9, *R*^2^ = 0.003, *p* = 0.54; Aitchison *F* = 1.35, *R*^2^ = 0.004, *p* = 0.09) and samples from the same run did not cluster together (Supplementary Figure S1). Each run always had the same number of samples and the number of reads per sample was extremely consistent across runs.

### Bioinformatic processing of PacBio CSS reads

Raw PacBio reads were converted to HiFi reads for each sample using the SMRT Analysis software (v.11.0.0.146107). The resulting reads underwent quality trimming, denoising, dereplication, and chimera removal using the dada2 plugin within QIIME 2 (14), following the protocol outlined by Anderson et al. (15). Specifically, reads shorter than 1,300 bp and longer than 1,600 bp after adapter trimming were removed, the pseudo-pooling method was used for denoising, the maximum number of expected errors was set to 3, and the pooling method was used for chimera detection. After processing with DADA2, a table of ASV counts for each sample was produced. Samples average 7,026 sequences post-processing.

For ASV taxonomic assignment, we employed the Naive Bayes trained sklearn classifier (16) within QIIME 2, utilizing our manually curated version of the Silva (v.138.1 NR99) reference database (17) as detailed in Anderson et al. (15) and in AnimalBiome’s pipeline for classifying PacBio full-length 16S rRNA HiFi reads.^1^ The confidence threshold was set to 0.7, which has been shown to perform the best for the naive Bayes classifier (16). These taxonomic labels were further refined using stringent VSEARCH (18) classification also within QIIME2. This dual hybrid approach enabled greater specificity in the taxonomic labels (e.g., if an ASV was assigned Genus-level classification by sklearn but VSEARCH assigned it to Species with 100% confidence, then we retained the species-level call) and confidence in these assignments. ASVs not classified at the Family level were filtered from the dataset. For ASVs unclassified at the species level, we appended “unclassified” to their existing taxonomic label.

The table of ASV counts was aggregated at the species level and imported into the R statistical software program (v.4.3.0) for subsequent statistical analyses and visualizations.

### Statistical analysis

The primary objective of this study was to identify the bacterial species comprising the core microbiome of dogs in the healthy reference set. To achieve this, we determined the prevalence of each bacterial taxon by dividing the number of samples containing that taxon by the total sample size. Additionally, we computed various descriptive statistics on taxon relative abundances including the mean, median, minimum (excluding 0 s), maximum, standard deviation, and percentiles (0.025, 0.1, 0.9, 0.975). Bacterial species with a prevalence of at least 33.333% and mean relative abundance >0.5% were designated as part of the core. This allowed us to capture highly prevalent taxa, as well as highly abundant taxa, and extract patterns of insight that are reflective of our study population. Additionally, because the core was being defined at the level of bacterial species and not broader bacterial genera, we deemed these thresholds appropriate. For statistical analysis, two core microbiome metrics were computed: (i) the number of core taxa, and (ii) the core microbiome sum, representing the proportion of the microbiome composed of core taxa.

The secondary objective was to identify host factors correlated with the core microbiome metrics, and microbiome alpha- and beta-diversity in this healthy reference set. Four types of statistical models were constructed: (i) a model that included sex & neuter status, age (years), and body weight (kg) as predictor variables, (ii) another model that specified diet as the sole predictor variable and excluded dogs with combined diets such as “Kibble & Raw,” (iii) a third model that had USA geographic region (West, Midwest, Northeast, South) as the only predictor, and filtered dogs residing in Canada or in unknown North American locations, and (iv) a fourth model that compared the fecal microbiomes of dogs in the reference set that are part AnimalBiome’s stool donor program with those that are not.

For both alpha- and beta-diversity analyses, microbiome data from dogs in the healthy reference set were subsampled to 3,500 reads per sample to account for uneven sequencing depth and minimize the risk of falsely detecting or rejecting group differences (19). This resulted in the exclusion of 24 samples. Although this sequence count cutoff might seem low compared to studies using Illumina short-read sequencing (150 or 250 bp amplicons), it is appropriate and robust for studies utilizing full-length 16S rRNA gene PacBio HiFi reads (1,600 bp), where throughput is lower but reads are longer. The longer sequences allow for a greater proportion of reads assigned to the species level compared to Illumina sequencing (11).

We computed three alpha-diversity metrics [Chao 1 Richness (log), Shannon Diversity, and Gini-Simpson’s index (1- Simpson’s Index)] using the *phyloseq* package (v.1.44.0) (20). Generalized additive models (GAMs) from the *mgcv* package (v.1.8–42) (21) correlated microbiome alpha-diversity or core microbiome metrics with the host factors of interest, as outlined in the above paragraph. In these GAMs, the two continuous predictors—age and body weight—were included as smooth terms, while all others were listed as linear terms. Hypothesis testing on the GAMs was conducted using Wald tests of significance from the *mgcv* package. Post-hoc comparisons were done with the *emmeans* (v.1.8.7) (22) and *multcomp* (v.1.4–23) (23) packages.

For beta-diversity analyses, rarefied microbiome data at the species level were converted to proportions for Bray-Curtis distances or applied a Center Log Ratio transformation for Aitchison distances. Both types of distances were estimated with the *phyloseq* package. Permutational Multivariate Analyses of Variance (PERMANOVAs) from the *vegan* package (v.2.6–4) (24) assessed the marginal effects of host predictor variables on Bray-Curtis and Aitchison distances, following the four models described earlier in this section. Post-hoc comparisons were done with the *pairwise Adonis* package (v0.4.1) (25) which employs Tukey tests.

Lastly, differential abundance testing was done with the *LinDA* package (v0.1.0) (26) to identify the bacterial species that were enriched in the fecal microbiomes of dogs according to their sex-neuter status, age, body weight, diet, or geographic region. The prevalence cutoff was set to 20%, winsorization cutoff (quantile) to 0.97, alpha to 0.05, and p.value adjustment as “FDR.” All figures included in this manuscript were constructed with the *ggplot2* package (v.3.4.2) (27).

One additional statistical test was conducted that compared the fecal microbiomes of dogs in the healthy reference set (*n* = 286), with those from dogs receiving bacterial probiotics (*n* = 86) that would otherwise qualify to be in the healthy reference set. Statistical models in the forms of GAMs or PERMANOVAs specified rarefied microbiome alpha-diversity, beta-diversity, or core microbiome metrics as the dependent variable and bacterial probiotic intake (True/False) as the independent variable.

## Results

### Characteristics of healthy dogs

The dogs that formed part of the reference set (*N* = 286, Table 1) were verified to be healthy (*N* = 56) or were presumed to be healthy (*N* = 230), and had a median age of 4 yrs., median body weight of 21.2 kg, and median body condition score of 5. They tended to be medium- to large-size dogs, with 40% found in the 10–25 kg category and another 34% placed in the 25–45 kg category (Table 1). 70% of dogs were spayed or neutered (36% females, 41% males). The most common breeds were Australian and German Shepherds (12%), Goldendoodles and Poodles (10%), Golden Retrievers and Labrador Retrievers (10%), and Terriers (8%). About 30% of dogs were fed only dry kibble, another 27% ate only raw food, and 16% were fed only cooked food, with the remaining dogs (27%) consuming a combination of the aforementioned diets (Table 1). 70% of dogs in the reference set resided in known locations in the USA (39% came from the West coast, 13% from the Northeast, 10% from the South, and 8% from the Midwest), 2% resided in Canada, and for the remaining participants (28%), their specific North American location or region was unknown.

**Figure 1:**
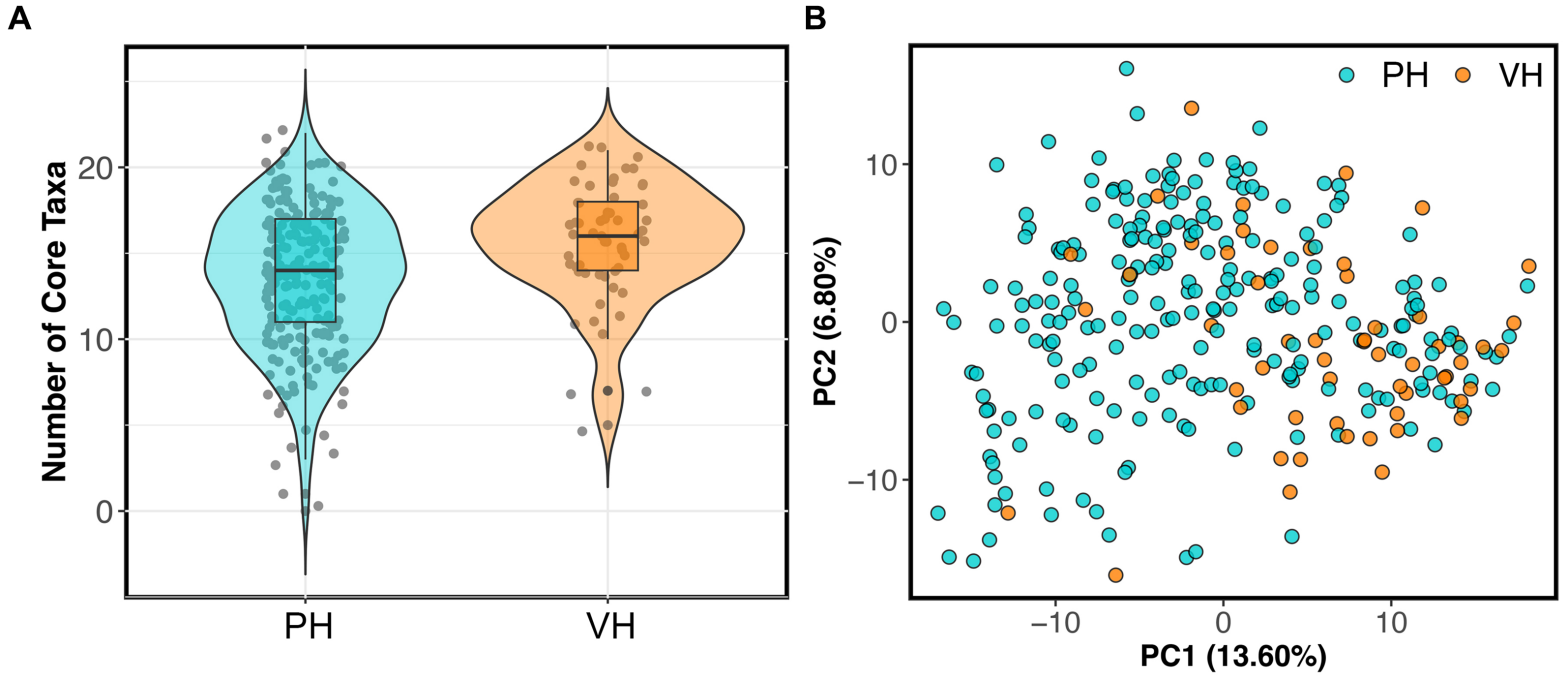
Fecal microbiomes differ between verified healthy and presumed healthy dogs. Dogs in the healthy reference set were categorized as “verified healthy” (VH) if they were verified to be healthy via medical records, veterinary visits, and monthly parasite screenings, or “presumed healthy” (PH) if they were not and were reported to be healthy by their owners. **(A)** Number of core taxa for verified healthy vs. presumed healthy dogs. **(B)** PCoA ordination based on Aitchison distances showing the clustering of verified healthy vs. presumed healthy microbiomes.

As mentioned, 56 dogs in the reference set (20% of dataset) were part of AnimalBiome’s stool donor program and thus, were verified to be healthy via medical records, veterinary visits, and monthly monitoring of parasites and pathogens. This presented a unique opportunity to examine how the microbiomes of clinically validated healthy dogs compared to those from presumed healthy dogs. We found that the microbiomes of stool donors were richer than the microbiomes of presumed healthy dogs (GAM LRT Chao 1 Richness *F* = 3.67, *p* = 0.056; Shannon Diversity *F* = 1.5, *p* = 0.22; Gini-Simpson index *F* = 1.83, *p* = 0.038). Donors had a slightly greater number of core taxa (x̄: 15.64 taxa) than did non-stool donors (x̄: 13.29 taxa) (Figure 1A); donors also had more of their microbiome comprised by core taxa (80.8% of their microbiome) than did non-stool donors (73.9% of their microbiome) (GAM LRT Core taxa number *F* = 14.96, *p* = 0.001; Core Sum *F* = 4.13, *p* = 0.04). Lastly, donors have marginally distinct fecal microbiomes from those of apparently healthy dogs (PERMANOVA Bray-Curtis *R*^2^ = 0.019, *p* = 0.001; Aitchison *R*^2^ = 0.024, *p* = 0.001) (Figure 1B). This implies that, as anticipated, some presumed healthy dogs may not possess the same level of health as perceived by their owners, or that the screening criteria for clinically validated dogs may favor a narrower spectrum of microbiome compositions.

### Core fecal microbiome of healthy dogs

**Figure 2:**
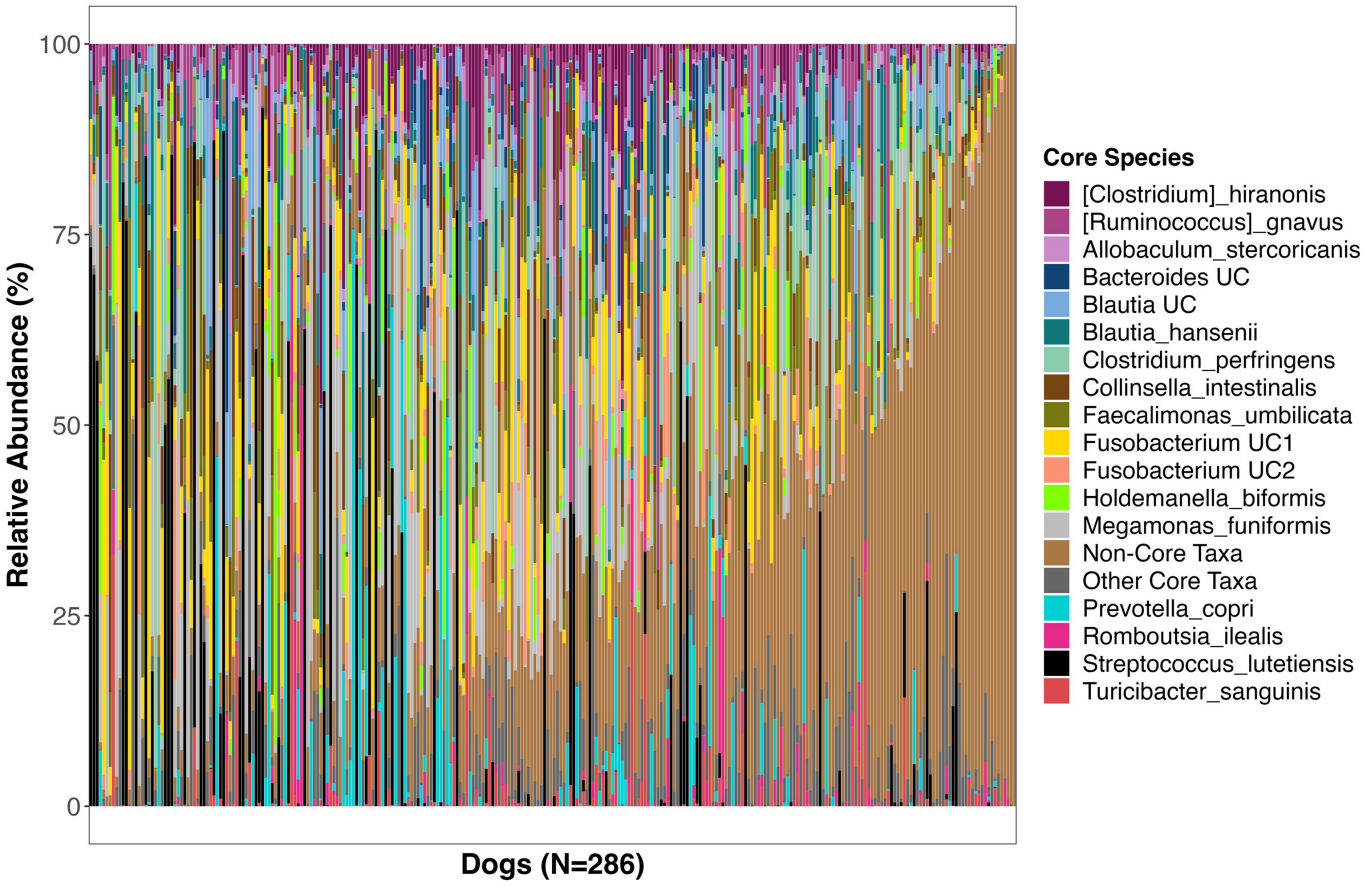
Relative abundances of bacterial species comprising the core microbiome in healthy dogs. These bacterial species were found in at least 33% of dogs at a mean relative abundance >0.5%. UC, unclassified.

Twenty-three bacterial species formed part of the core microbiome in verified healthy and presumed healthy dogs (Table 2, Figure 2, and Supplementary Table S1). These were taxa that were found in at least 1 of 3 dogs that formed part of the healthy reference and had a mean relative abundance >0.5% across the dataset. This was an appropriate cutoff for a core defined at the bacterial species-level. Additionally, given the large variability found in the surveyed canine microbiomes, a stricter cutoff would not have been informative or representative of our data.

**Table 2:** Twenty-three bacterial species comprise the core microbiome in healthy dogs.

Bacterial species	Prev.	Mean	Median	Min*	Max	Stdev	Low10pct	Pct50	High90pct	High97.5pct
*Blautia hansenii*	0.867	3.656	1.008	0.045	50.525	7.048	0.000	1.008	10.260	22.576
*Ruminococcus gnavus*	0.846	3.101	0.970	0.049	68.980	6.746	0.000	0.970	7.947	23.084
*Faecalimonas umbilicata*	0.832	4.430	0.874	0.020	50.997	8.311	0.000	0.874	12.328	28.769
*Blautia* UC	0.797	3.948	1.287	0.022	51.346	7.174	0.000	1.287	11.727	29.979
*Fusobacterium* UC 1	0.766	6.558	2.259	0.032	46.319	9.333	0.000	2.259	18.702	34.324
*Collinsella intestinalis*	0.745	4.877	1.714	0.037	46.192	7.514	0.000	1.714	14.671	26.654
*Megamonas funiformis*	0.671	11.270	1.441	0.024	80.080	17.837	0.000	1.441	37.305	62.057
*Fusobacterium* UC 2	0.654	2.520	0.283	0.017	41.563	5.863	0.000	0.283	6.423	22.214
*Peptacetobacter hiranonis*	0.654	3.274	0.475	0.031	47.203	6.327	0.000	0.475	9.412	21.417
*Blautia marasmi*	0.636	0.642	0.225	0.018	7.525	1.104	0.000	0.225	2.038	3.848
*Lachnoclostridium* UC	0.605	0.758	0.135	0.019	20.325	2.010	0.000	0.135	1.729	5.744
*Blautia caecimuris*	0.573	0.864	0.093	0.031	13.586	1.917	0.000	0.093	2.354	7.551
*Clostridium perfringens*	0.510	5.328	0.040	0.032	95.001	12.995	0.000	0.040	18.875	46.342
*Romboutsia* UC	0.493	1.035	0.000	0.029	30.672	2.979	0.000	0.000	2.613	11.264
*Blautia glucerasea*	0.462	1.083	0.000	0.020	36.227	3.388	0.000	0.000	2.378	11.972
*Turicibacter sanguinis*	0.441	1.551	0.000	0.032	52.813	5.268	0.000	0.000	3.501	14.519
*Romboutsia ilealis*	0.416	1.300	0.000	0.038	55.220	4.735	0.000	0.000	2.974	14.949
*Bacteroides* UC	0.409	1.930	0.000	0.025	48.088	5.481	0.000	0.000	4.909	18.855
*Escherichia coli*	0.402	1.030	0.000	0.025	48.403	3.760	0.000	0.000	2.478	10.002
*Holdemanella biformis*	0.395	2.093	0.000	0.032	73.689	6.319	0.000	0.000	6.181	15.669
*Allobaculum stercoricanis*	0.392	1.329	0.000	0.024	38.850	4.160	0.000	0.000	3.209	11.478
*Prevotella copri*	0.350	3.331	0.000	0.034	75.757	8.934	0.000	0.000	11.139	30.045
*Streptococcus lutetiensis*	0.336	9.444	0.000	0.020	91.673	22.138	0.000	0.000	47.421	78.048

UC, unclassified; prev., prevalence; pct, percentile.

*The minimum value excludes 0 s; true minimum value is 0 which is not informative.

The bacterial core species with the highest prevalence (>60%) were: *Blautia hansenii*, *Ruminococcus gnavus*, *Faecalimonas umbilicata*, unclassified *Blautia*, unclassified *Fusobacterium*, *Collinsella intestinalis*, *Megamonas funiformis*, *Peptacetobacter hiranonis*, *Blautia marasmi*, and unclassified *Lachnoclostridium* (Table 2). Other bacterial species that were found at slightly lower prevalences but were also part of the core included *Escherichia coli*, *Prevotella copri*, *Romboutsia ilealis*, *Sutterella stercoricanis*, *Turicibacter sanguinis*, and *Streptococcus lutetiensis*. Of the 23 core bacterial taxa, the ones found at the highest relative abundances in dogs were *Megamonas funiformis* (11.2% mean relative abundance), *Streptococcus lutetiensis* (9.4%), unclassified *Fusobacterium* (6.5%), *Clostridium perfringens* (5.3%), *Collinsella intestinalis* (4.8%), and *Faecalimonas umbilicata* (4.4%) (Figure 2).

On average, the core microbiome represented the overall canine microbiome well and made up 75% of the sequences detected in a sample. However, the presence and relative abundances of core taxa were widely variable among dogs, with some dogs having some core taxa but not others. Interestingly, 10% of dogs in the dataset appeared to harbor a completely different set of bacteria that were not part of the core (Supplementary Figure S2). These dogs instead harbored high abundances of *Bacteroides vulgatus*, *Bifidobacterium pseudocatenulatum*, and *Lactobacillus acidophilus*, among others (Supplementary Figure S2).

### Effects of sex-neuter status, age, body weight, diet, and geography on the microbiome

**Figure 3:**
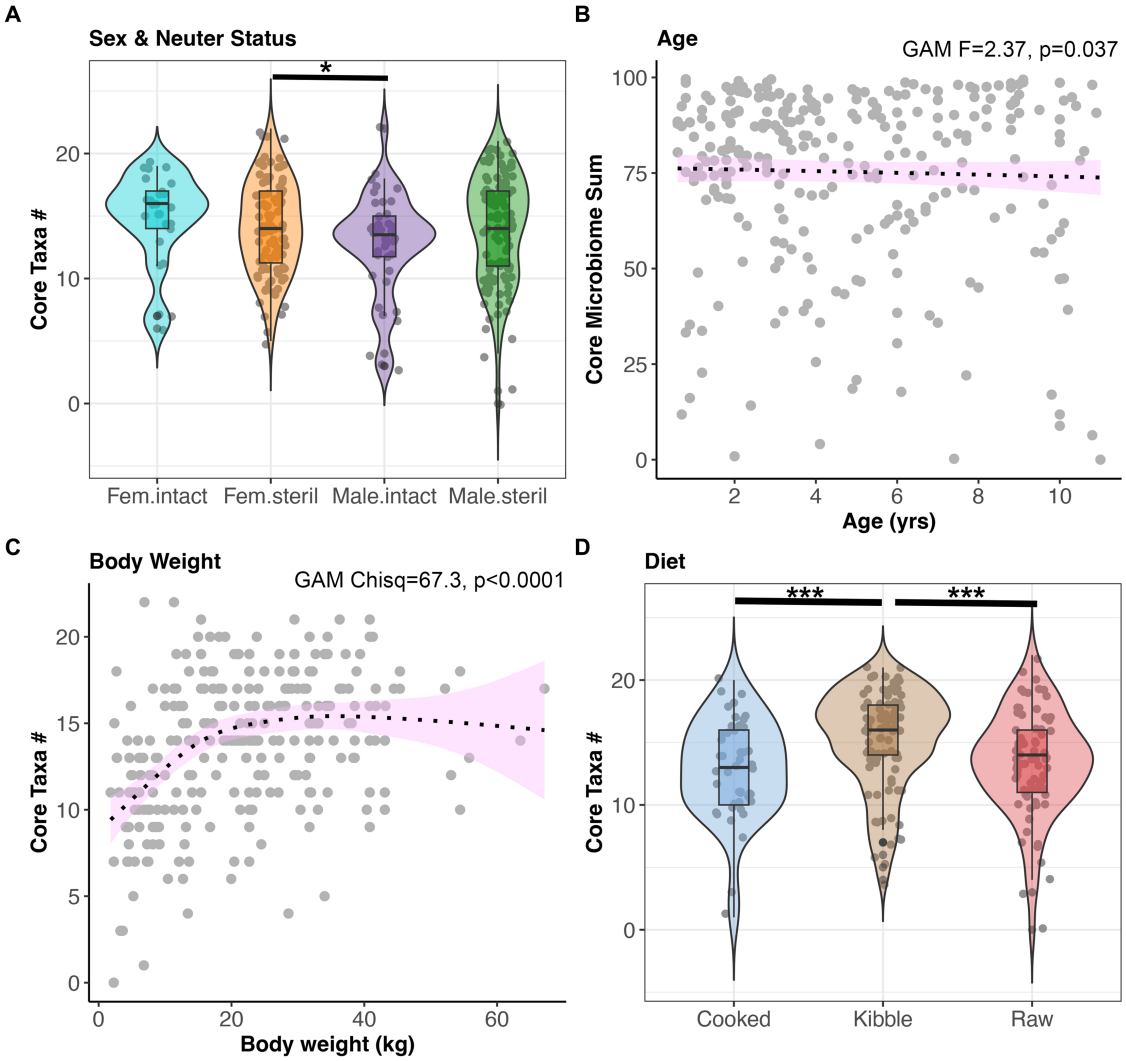
The number of core taxa present in canine fecal microbiomes varies with sex & neuter status, age, body weight, and diet. All dogs that were part of the dataset had BCS 4–6, and no history of antibiotics, medications, bacterial probiotics, and physical conditions. **(A)** Number of core taxa (max 23) for each Sex-Neuter category. **(B)** Proportion of the microbiome made up of core taxa regressed against age, with smooth curve overlaid to illustrate relationship between x and y. **(C)** Number of core taxa plotted against body weight in kg. **(D)** Number of core taxa for each diet category. *p < 0.05, **p < 0.01, ***p < 0.0001.

Next, we examined the impact of sex-neuter status, age (yrs), body weight (kg), diet, and geographic region on the core microbiome and on microbiome alpha-diversity and beta-diversity of presumed healthy and verified healthy dogs. Overall, the fecal microbiomes of sterilized females had marginally more core taxa (x̄:14.2) than the microbiomes of intact males (x̄:12.72) (GAM Tukey test *p* < 0.05, Figure 3A, and Supplementary Table S2). Dogs of intermediate body weights had more core taxa than small or large dogs (GAM *p* < 0.05, Figure 3C, and Supplementary Table S2). Dogs fed kibble tended to have slightly more core taxa in their microbiomes (x̄:15.1) than dogs fed raw food (x̄:13.3) or cooked food (x̄:12.9) (GAM Tukey test *p* < 0.05) (Figure 3D). However, the total percentage of the fecal microbiome composed of core taxa was not associated with sex & neuter status, body weight, or diet. This core metric was significantly associated with age; there was a modest decline in the proportion of the microbiome made up of core taxa as the dog aged (GAM *p* < 0.05) (Figure 3B and Supplementary Table S2). Lastly, dogs residing in different geographic regions within the USA did not differ significantly in the number or sum of their core microbiome taxa.

**Figure 4:**
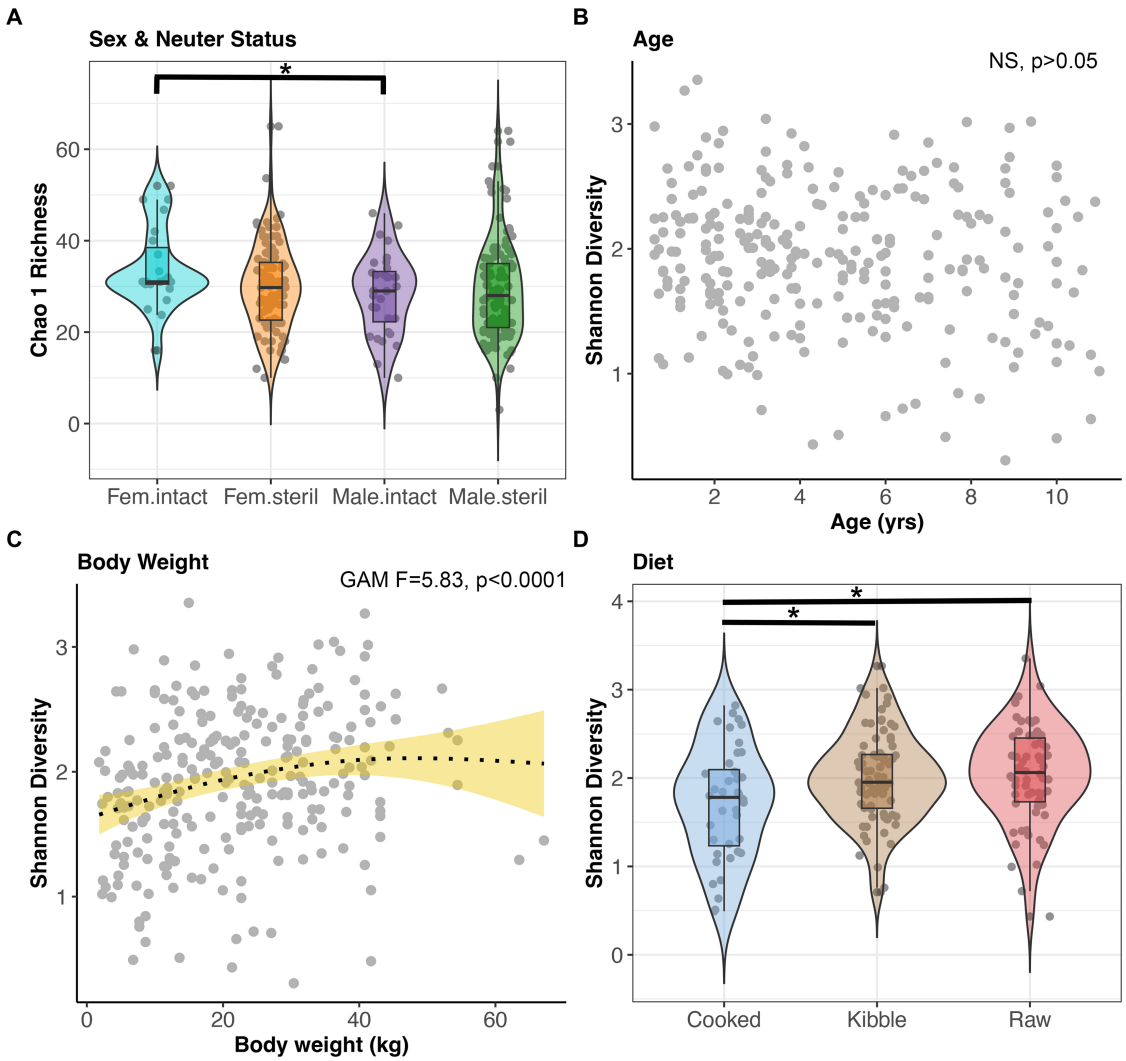
Microbiome alpha-diversity varies with dog sex & neuter status, age, body weight, and diet. **(A)** Chao 1 Richness by Sex-Neuter status (Female intact, Female sterilized, Male intact, Male sterilized). **(B,C)** Shannon diversity plotted against age in years or body weight in kg, with smooth curve overlaid to illustrate relationship between x and y. **(D)** Shannon diversity for each diet category. *p < 0.05, **p < 0.01, ***p < 0.0001.

Microbiome alpha-diversity was significantly correlated with all host factors examined except age and geographic region (GAM, *p* < 0.05, Figures 4A–D, and Supplementary Table S3). Specifically, the microbiomes of intact females (x̄ Chao1 Richness: 33.94) were more diverse than the microbiomes of sterilized males (x̄: 29.29) (Tukey test *p* < 0.05, Figure 4A). Microbiome alpha-diversity was highest in dogs of intermediate body weights (20–45 kg) (GAM *p* < 0.05, Figure 4C). Dogs that consumed a cooked food diet (x̄ Shannon Diversity: 1.71) had less diverse fecal microbiomes than dogs that consumed kibble (x̄: 2) or raw food (x̄: 2.04) (Tukey test *p* < 0.05, Figure 4D).

**Figure 5:**
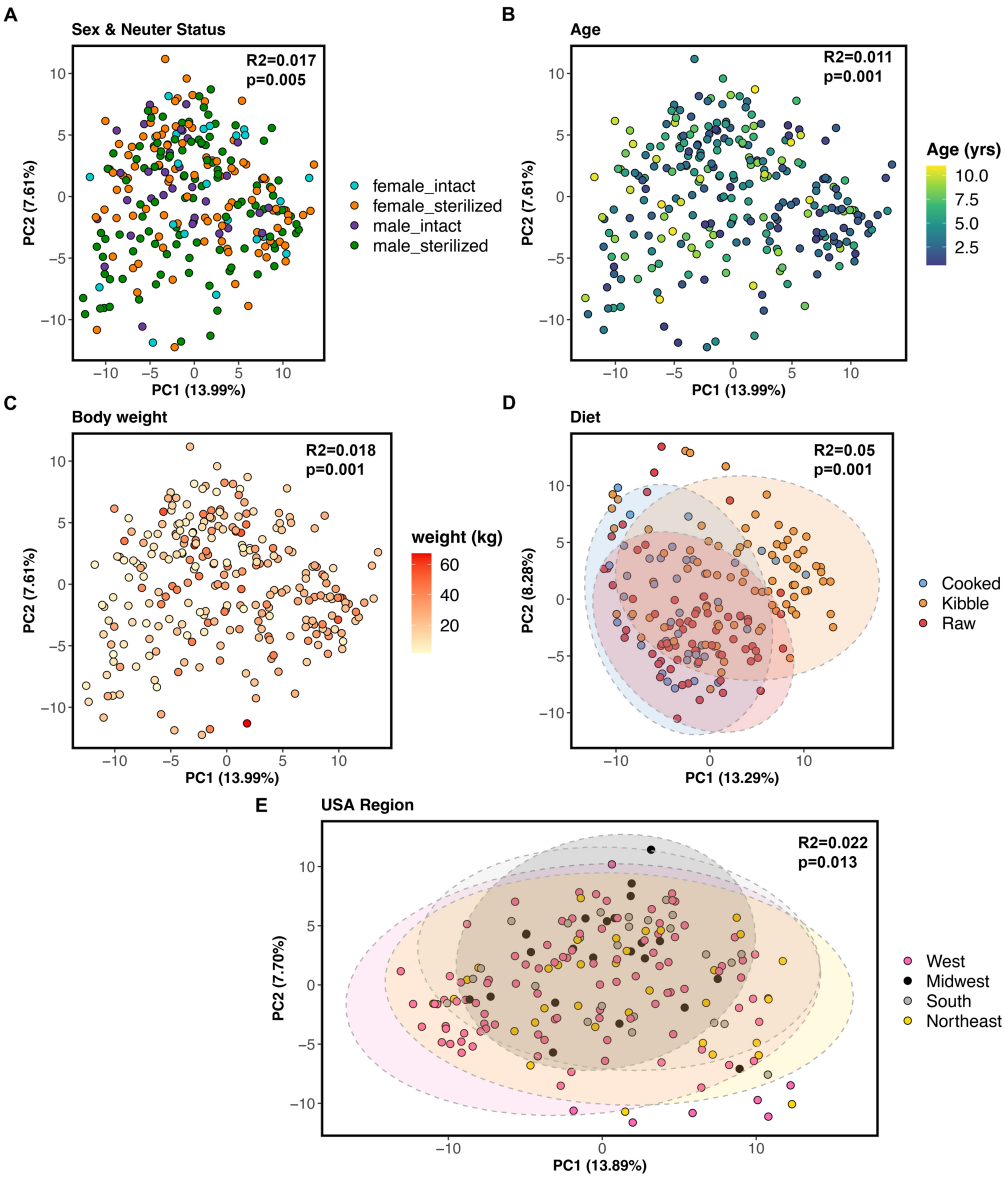
Host correlates of fecal microbiome beta-diversity in healthy dogs. PCoA ordinations based on Aitchison distances color coded by **(A)** sex & neuter status, **(B)** age (yrs), **(C)** body weight (kg), **(D)** diet, or **(E)** geographic region. PERMANOVA *R*^2^ and *p*-values are shown.

All of the factors examined in this study significantly predicted fecal microbiome beta-diversity (PERMANOVAs *p* < 0.05, Figures 5A–E, and Supplementary Table S4), but the effect sizes were low. A dog’s diet accounted for the largest variance in the microbiome (5%), followed by geographic region (2.2%), body weight (1.8%), sex and neuter status (1.1%), and age (1.1%) (Supplementary Table S4). In an ordination plot, none of the host factors examined formed defined clusters which suggests that fecal microbiomes of dogs are likely influenced by a multitude of host factors. No one host factor alone can predict the composition of canine fecal microbiomes, even in healthy individuals that do not have any physical conditions, were not taking medications, and were of similar body conditions.

Nevertheless, we conducted post-hoc comparisons to determine which groups of dogs differed in their beta-diversity. The microbiomes of dogs fed kibble were modestly different from those of dogs fed raw food (Bray-Curtis Tukey test *F* = 6.61, *R*^2^ = 0.043, *p* = 0.001; Aitchison Tukey test *F* = 7.01, *R*^2^ = 0.046, *p* = 0.001) or cooked food (Bray-Curtis Tukey test *F* = 3.89, *R*^2^ = 0.031, *p* = 0.001; Aitchison Tukey test *F* = 5.42, *R*^2^ = 0.043, *p* = 0.001). Regarding geographic region, minor differences were found between the microbiomes of dogs living in the western USA and dogs living in the Midwest (Bray-Curtis Tukey test *F* = 1.63, *R*^2^ = 0.013, *p* = 0.05) or Northeast (Aitchison Tukey test *F* = 1.57, *R*^2^ = 0.011, *p* = 0.05) (Figure 5E). The microbiomes of intact females were slightly different from the microbiomes of sterilized males (Aitchison Tukey test *F* = 1.75, *R*^2^ = 0.013, *p* = 0.02).

One additional statistical comparison was conducted to determine whether the intake of bacterial probiotics impacted the microbiome. For this, the fecal microbiomes of dogs in the healthy reference set (*n* = 286) were compared with the fecal microbiomes of dogs that received bacterial probiotics (*n* = 86) and would otherwise qualify to be part of the healthy reference set. The two groups did not differ in terms of their core species (GAM LRT Core sum *F* = 0.004, *p* = 0.94; Core number *F* = 0.92, *p* = 0.33) or alpha-diversity (GAM LRT Chao 1 Richness *F* = 0.218, *p* = 0.641; Shannon Diversity *F* = 0.078, *p* = 0.78; Gini-Simpson index *F* = 0.002, *p* = 0.964). The two groups differed marginally in terms of their beta-diversity (PERMANOVA Bray-Curtis *R*^2^ = 0.003, *p* = 0.19; Aitchison *R*^2^ = 0.004, *p* = 0.052).

### Identifying bacterial species that are enriched in the fecal microbiomes of dogs

Given that microbiome beta-diversity was moderately associated with sex & neuter status, age, body weight, diet, and geographic region, we sought to identify the bacterial species that could underlie those differences.

**Figure 6:**
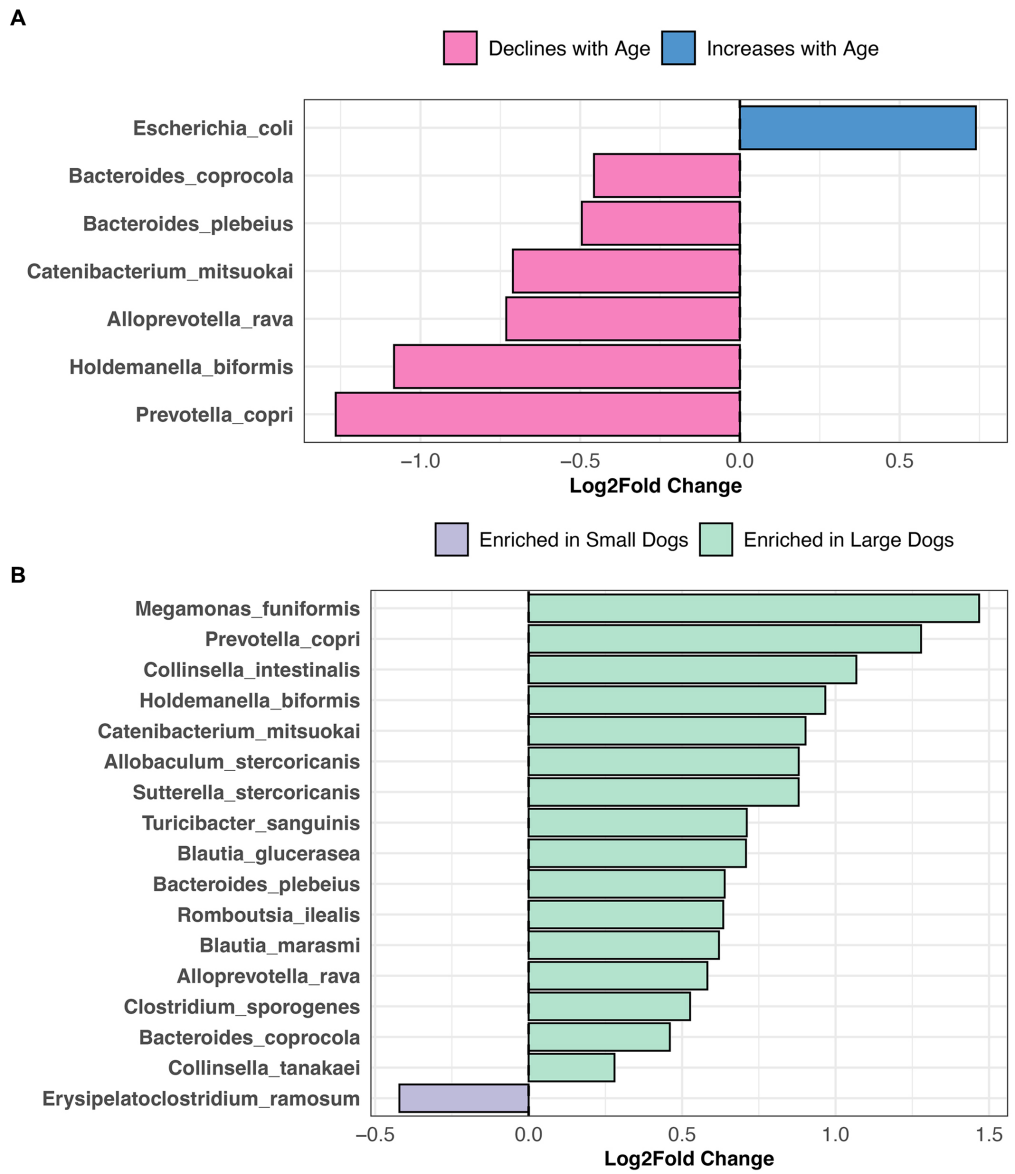
Bacterial species enriched in the microbiomes of young dogs and large dogs. Results from LinDA differential abundance analyses performed at the bacterial species level. **(A,B)** The LinDA model included age (yrs) and body weight (kg) as continuous variables, and sex & neuter status as a categorical variable, though no statistically significant taxa emerged for the categorical predictor.

Differential abundance testing revealed that the abundances of six bacterial species, among them *Prevotella copri*, *Alloprevotella rava*, and *Bacteroides coprocola* decline with age, but the opposite was true for *Escherichia coli* abundances (LinDA p.adjusted <0.05, Figure 6A, and Supplementary Table S5). Sixteen bacterial species were more abundant in the fecal microbiomes of large dogs compared to smaller dogs. These species were *Megamonas funiformis*, *Collinsella intestinalis*, *Sutterella stercoricanis*, *Turicibacter sanguinis*, *Bacteroides plebeius*, and *Collinsella tanakaei*, among others (LinDA p.adjusted <0.05, Figure 6B, and Supplementary Table S5). Differential abundance testing was not able to single out any particular bacterial species as varying significantly between intact females and sterilized males (LinDA p.adjusted >0.05, Supplementary Table S5).

**Figure 7:**
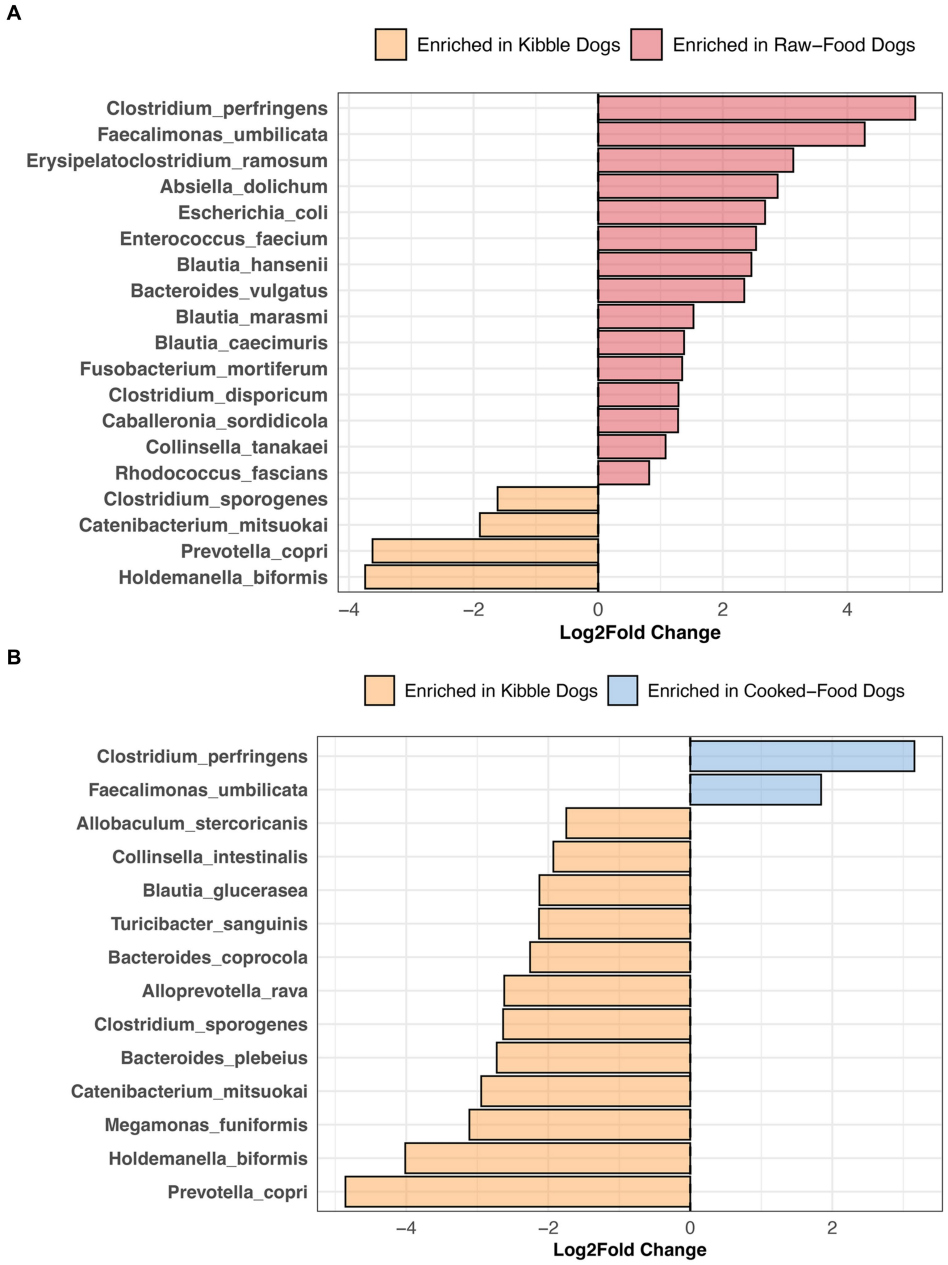
Bacterial species enriched in the microbiomes of dogs according to their diet category. Results from LinDA differential abundance analyses performed at the bacterial species level. The LinDA model included diet category (Kibble, Raw, or Cooked) as the dependent variable. **(A)** Dogs fed Kibble vs. Dogs fed Raw food. **(B)** Dogs fed Kibble vs. Dogs fed Cooked food. No bacterial taxa were differentially abundant between dogs fed cooked food compared to raw food, hence why these plots are not displayed.

Compared to dogs fed only kibble, the fecal microbiomes of dogs fed raw food were enriched in 15 bacterial species, including *Bacteroides vulgatus*, *Caballeronia sordidicola*, *Enterococcus faecium*, *Erysipelatoclostridium ramosum*, three *Blautia* sp. and two *Clostridium* sp. (LinDA p.adjusted <0.05, Figure 7A, and Supplementary Table S6). A diet of only kibble enriched for *Collinsella intestinalis*, *Turicibacter sanguinis*, *Megamonas funiformis*, *Holdemanella biformis*, and *Prevotella copri*, among others (LinDA p.adjusted <0.05, Figures 7A,B). Dogs fed cooked food had an overrepresentation of two bacterial species: *Faecalimonas umbilicata* and *Clostridium perfringens* compared to dogs fed kibble (LinDA p.adjusted <0.05, Figure 7B). No differentially abundant bacteria taxa were detected between the fecal microbiomes of dogs that consumed cooked food compared to raw food (LinDA p.adjusted >0.05).

Lastly, dogs residing in the western USA tended to harbor larger abundances of *Enterococcus faecium* and *Escherichia coli* than dogs that lived in the Northeast region of the USA (LinDA p.adjusted <0.05). No differences in fecal bacterial abundances were identified between dogs living in western USA and dogs living in the Midwest.

## Discussion

To date, knowledge of the bacterial species comprising the core fecal microbiome in healthy dogs is limited. Here, we used full-length 16S rRNA gene sequencing to gain novel insights into the bacterial species residing in the healthy canine gut. Specifically, we profiled the fecal microbiomes of 286 total healthy dogs—56 verified to be healthy and 230 presumed to be healthy—and identified a core microbiome comprising 23 bacterial species, which included both well-established beneficial taxa like *Peptacetobacter hiranonis* and those belonging to genera traditionally associated with pathogenicity, such as *Escherichia coli* and *Streptococcus lutetiensis*. Host factors such as diet, age, body weight, sex & neuter status, and geographic region predicted microbiome variation.

It is important to emphasize that our study only evaluated a handful of host factors but other factors such as lifestyle, access to the outdoors, exercise frequency, and breed could also be significantly impacting microbiome variation. Additionally, our reference set of dogs all lived in homes in North America and were selected from a larger pool of dogs based on our chosen criteria. The study’s findings could change if the selection criteria are adjusted. We encourage future studies to examine whether the same patterns are observed for dogs in other continents or dogs residing in different living environments.

Nevertheless, even with these limitations, our study provides novel information regarding the composition and variation of canine fecal microbiomes, and demonstrates the utility of full-length 16S rRNA gene sequencing in microbiome research.

### Core microbiome species of healthy canines

Across 286 verified healthy or presumed healthy dogs, a core group of 23 bacterial species was detected, among them *Megamonas funiformis*, *Peptacetobacter hiranonis*, *Blautia hansenii*, *Escherichia coli*, *Turicibacter sanguinis*, *Prevotella copri*, *Sutterella stericoris*, *Streptococcus lutetiensis*, and unclassified *Fusobacterium.*

The core species belong to genera commonly found in the fecal microbiomes of dogs and cats. Likewise, in a dataset of 96 dogs from 9 different breeds, the most abundant bacterial genera were *Fusobacterium*, *Bacteroides*, *Prevotella*, *Blautia*, and *Lactobacillus* (28). Similarly, another study focusing on Maltese, Miniature Schnauzers, and Poodles found *Lactobacillus*, *Megamonas*, *Streptococcus*, *Blautia*, *Prevotella*, and *Escherichia* as the predominant bacterial groups (29).

However, much less is known beyond genus-level, as few canine microbiome studies have reported findings at a finer level of resolution. A recently published study did find that *Streptococcus lutetiensis*, *Collinsella intestinalis*, *Peptacetobacter hiranonis*, *Turicibacter sanguinis*, and *Blautia hansenii* were predominant in the fecal microbiomes of dogs receiving a low protein, low fiber diet and yeast probiotic (30). Another study surveying the microbiomes of 78 healthy dogs using shotgun metagenomic sequencing and qPCR reported the following three bacterial species as being part of the core (>78% prevalence): *Ruminococcus gnavus*, *P. hiranonis*, and *P. copri* (31). The remainder of the core was not defined at the species-level but contained genera represented in our dataset, e.g., *Blautia*, *Streptococcus*, and *Fusobacterium* (31). Their core also contained *Bifidobacterium* and *Lactobacillus* which were more rare in our dataset, likely due to differences in sequencing technologies and study populations.

Among the bacterial species detected in the core, *Peptacetobacter hiranonis*, is known for its beneficial effects on canine gut health. These anaerobes are the main group of microbes that dehydroxylate primary bile acids (PBAs) into secondary bile acids (SBAs) in the mammalian gut (32, 33). PBAs facilitate the emulsification of dietary fats and aid in the digestion and absorption of lipids; however, when conjugated, they can also be toxic to bacteria (34, 35). SBAs are involved in lipid metabolism, cell autophagy and immune system activation (36–38), but when low, are a biomarker of dysbiosis. Dogs with chronic enteropathies, inflammatory bowel disease, or antibiotic-induced dysbiosis (9, 39–41) have substantially reduced abundances of *P. hiranonis* and lower levels of SBAs compared to healthy dogs. Restoration of *P. hiranonis* abundances via the administration of prebiotics, synbiotics, or fecal transplants may restore bile acid metabolism and decrease dysbiosis (40, 42).

*Prevotella copri* is another beneficial microbe known for its role in producing short-chain fatty-acids (SFCAs) in the gut, primarily succinate, acetate, and formate from the fermentation of carbohydrates (43–45). SCFAs serve as rich energy sources for other microbes or for host colonocytes (46), exhibit anti-inflammatory effects (47), and protect the mucosal intestinal barrier (48). Dogs with acute hemorrhagic diarrhea syndrome have lower abundances of *P. copri* compared to healthy controls, and administering colonoscopic fecal microbiota transplants (FMTs) to these dogs increases their *P. copri* levels (49). In mice, administration of *P. copri* via oral gavage improves glucose tolerance in individuals consuming a high-fiber, low-fat diet (50).

Less is known about other canine core microbiome species such as *Turicibacter sanguinis*. In mice, the absence of *Turicibacter* spp. in the gut is associated with increased susceptibility to severe *Citrobacter rodentium* infection and colonization with *T. sanguinis* provides protection from disease (51). Shotgun metagenomic analyses suggest that *T. sanguinis* may also be involved in bile acid, lipid, and steroid metabolism and the regulation of systemic triglyceride levels (52, 53). Dogs with gastrointestinal diseases including chronic enteropathy, acute and chronic diarrhea, and inflammatory bowel disease have lower abundances of *Faecalibacterium*, *Turicibacter*, *Blautia*, *Fusobacterium*, and *P. hiranonis*, and higher abundances of *Escherichia coli* compared to healthy dogs (54). However, more research is needed to uncover the potential impacts of *T. sanguinis* on canine health and the microbiome.

The presence of *Escherichia coli* in the gut is not always associated with disease. More than 700 serotypes of *E. coli* have been identified across humans, domestic mammals, and wild mammals (55, 56), and only a small fraction contain the virulence factors (57) that cause infections. In a recent study, 38 *E. coli* isolates were recovered from the fecal samples of healthy dogs, and these formed a distinct phylogenetic group from *E. coli* isolates recovered from dogs with diarrhea (58). Another study reported over 69 unique *E. coli* isolates collected from the feces of 183 healthy dogs (59). While some of the isolates demonstrated resistance against antibiotics, this in itself does not imply virulence or disease-causing potential. In the absence of clinical symptoms, *E. coli* appears to be a natural resident of the canine fecal microbiome and our work supports that. However, overgrowth of *E. coli* can indicate a microbiome imbalance, which could have functional consequences for the host.

It may be surprising that a *Streptococcus* species (*S. lutetiensis*) was prevalent in the microbiomes of presumed healthy dogs in our study. Pathogenic *Streptococcus* spp. (e.g., *S. equi* subsp. *zooepidemicus*) (60) are well documented but commensal (e.g., *S. canis*) and potentially beneficial Streptococci (e.g., *S. dentisani*) also exist (61). *S. lutetiensis* has been isolated from healthy dogs administered oligofructose and inulin (62), but is also found in high proportions in the microbiomes of dogs with lymphoma compared to healthy dogs (63). These findings suggest that the role of *S. lutetiensis* in canine health is complex and warrants further investigation. Significant taxonomic changes and genomic similarities among *Streptococcus* spp. add another layer of complexity. Taxonomic misidentifications have prompted researchers to place *S. lutetiensis* within the broader *Streptococcus bovis* group, alongside four other species of *Streptococcus* (64, 65). We encourage future work to isolate and characterize *S. lutetiensis* isolates from healthy dogs to broaden our understanding of their roles.

### Host correlates of microbiome variation in healthy canines

We detected a core group of bacteria in healthy dogs, but their abundances varied among individuals. Some of this variation was associated with host age, spay-neuter status, body weight, diet, probiotic use, and geographic region, although the effect sizes were low. Perhaps other factors that we did not examine (e.g., breed, exercise frequency, urban vs. rural, host genetics) could be accounting for variation. There could also be latent interactions among the host factors themselves (e.g., are intact dogs more likely to be raw-fed, and smaller dogs less likely to be exercised?), that could be complicating our findings. Yet another possibility is that the microbiome is complex and host-associated factors alone cannot capture its variation.

Age-related effects on the microbiome have been observed in companion animals. In healthy cats, aging reduces the number of core taxa in the fecal microbiome (66), similarly, in this study, the proportion of the microbiome composed of core taxa decreased with age. Microbiome beta-diversity also shifts with age. In Beagles, the microbiome differs between pre-weanling, weanling, young, aged, and senile individuals (67), particularly in the abundances of *Lactobacillus* spp. and *Bifidobacterium* spp. Another study conducted across 288 shelter cats found that older cats harbored greater abundances of *Clostridium baratii*, *Turicibacter* spp., and *Campylobacter* than younger cats (68).

Spay & neuter status was also significantly associated with microbiome variation. Differences were mainly detected between sterilized females (or males) and intact males (or females). These findings align with recent research that surveyed the fecal microbiomes of 132 dogs and reported distinct clustering between intact individuals of both sexes and sterilized individuals of both sexes (69). Sterilized female dogs also have vaginal microbiomes that are distinct from those of intact females (70).

Our study found evidence that body weight was predictive of microbiome variation, with diversity highest at intermediate weights. Similarly, a meta-analysis of body size on canine digestive physiology reported a negative correlation between body weight and the relative abundances of *Proteobacteria* for some of the studies they examined (71). Broader patterns of microbiome variation with canine and feline body condition (72–74) and obesity (75–77) have also been reported.

We found that slight differences were detected between the microbiomes of dogs in the western USA and dogs in the Midwest or Northeast, echoing findings from a study (78) that surveyed the fecal microbiomes of 192 dogs from the Western and Midwestern parts of the USA. The study attributed microbiome patterns to regional differences in the degree of urbanization and diversity of pet food available. Geographic disparities in the lifestyles of owners and their pets, noise pollution, weather, and socialization practices could also underpin these differences.

Interestingly, we observed that the fecal microbiomes of dogs in the reference set were marginally distinct from those of dogs that received bacterial probiotics. Bacterial probiotics have been shown to influence microbiome composition in humans with obesity (79), cats or dogs with chronic diarrhea (80, 81), and healthy dogs (82–84), but they have also been shown to have no effect (85–88).

### Influence of diet on the canine gut microbiome

Our work showed that diet accounted for the most variation in the microbiome. Particular differences were found between dogs fed kibble and dogs fed raw food or cooked food. The three diets vary significantly in their nutrient composition, and bioavailability of these nutrients (89) which inevitably shapes the fecal microbiome and favors microbes that are able to utilize the digested components. Kibble diets for example, comprise a blend of cereal grains and meats, and contain lower levels of protein and fat compared to some raw meat-based diets, also known as Biologically Appropriate Raw Food (BARF) diets. RMBDs consist primarily of uncooked meat, although fiber-rich ingredients may be added. To complicate things even further, even within a diet category such as kibble, nutrient profiles can vary significantly.

In our study, dogs fed RMBDs were enriched in *Clostridium perfringens*, *Bacteroides vulgatus*, *Enterococcus faecium*, *Caballeronia sordicola*, and *Collinsella tanakei*, among others compared to dogs that consumed kibble. Other studies have also reported higher abundances of *C. perfringens* in the fecal microbiomes of dogs fed a BARF diet compared to a commercial diet (90–92). *C. perfringens* are proteolytic bacteria adapted to breaking down protein into smaller components (93); hence, their abundance in high-protein diets. In broiler chickens, the levels of *C. perfringens* present in the ileum and cecum increase as the level of crude protein in their fishmeal-based diet also increases (94).

*Enterococcus faecium* and *Bacteroides vulgatus* were also enriched in the fecal microbiomes of dogs consuming RMBDs compared to kibble. Some strains of *E. faecium* (e.g., SF68) have been recognized for their potential probiotic benefits in dogs, aiding in specific immune functions (95) and diarrhea prevention (96). Similarly, *B. vulgatus* may be potentially beneficial in the gut. *B. vulgatus* is known to produce the fatty acids acetate, propionate, butyrate, and lactate (97, 98). They possess bile acid hydrolases (99, 100) that deconjugate primary bile acids.

Dogs primarily fed kibble were enriched in *Prevotella copri*, *Catenibacterium mitsuokai*, *Holdemanella biformis*, *Megamonas funiformis*, and *Bacteroides coprocola*, among others. Similarly, the fecal microbiomes of dogs fed kibble were enriched in bacteria from the genus *Megamonas*, *Faecalibacterium*, and *Catenibacterium* compared to dogs fed raw food (101). The fecal microbiomes of captive red wolves on a kibble diet are enriched in *Catenibacterium mitsuokai*, *Holdemanella* spp., and *Prevotella* spp. compared to red wolves fed a whole meat, wild, or mixed diet (102). Although *Holdemanella biformis* has shown to ameliorate hyperglycemia and restore gluconeogenesis in obese mice (103), its effects in dogs remain unexplored.

Interestingly, differential abundance testing failed to identify any bacterial species that were significantly associated with a cooked food versus raw food diet, perhaps the impact of cooking may vary depending on the food type. A study noted that while the gut microbiomes of mice fed raw versus cooked meat exhibited similar microbiome compositions and functions (104), the opposite was observed for mice fed cooked versus raw tubers.

### Future directions

While our study provides valuable insights regarding the healthy canine fecal microbiome, many open questions remain. The impact of other host lifestyle variables and geographic regions outside of North America should be examined. Seasonal variations in the microbiome or the influence of factors such as time of day and host circadian rhythms could be interesting to study. Our study did not include a longitudinal component or assess the stability of the healthy canine microbiome, but this is a priority for our future investigations. Additionally, we did not conduct metagenomic sequencing, which could offer deeper insights into the functional capabilities of the microbiome but encourage other studies to pursue this avenue of research.

## Conclusion

Our approach leverages full-length 16S rRNA gene sequencing and offers novel species-level insights into the fecal microbiome of verified healthy and presumed healthy dogs. Our findings highlight the prevalence of bacteria such as *Peptacetobacter hiranonis*, *Prevotella copri*, *Escherichia coli*, and *Streptococcus lutetiensis* in the microbiome and their potential impact on gut health. Specific microbial cocktails containing some of these core bacterial species could be developed to support pet health. Additionally, we identified age, spay-neuter status, body weight, diet and geographic region as modest predictors of microbiome variation, contributing to our understanding of the factors possibly interacting with the fecal microbiome in dogs.

## Data Availability

These data were generated using a proprietary reference database that was created through curating public data. Due to its commercial value, raw PacBio Hifi sequences, sample metadata, and microbiome data are only available upon request for academic research by emailing the corresponding author, HG, holly@animalbiome.com. Tables showing the output from all statistical tests are in the Supplementary materials.

## Footnotes

^1^https://github.com/AnimalBiome/AB_FlexTax/tree/main
