# Supplementary material for: Species-level characterization of the core microbiome in healthy dogs using full-length 16S rRNA gene sequencing
Source: Front Vet Sci. 2024 Sep 2;11:1405470. doi: 10.3389/fvets.2024.1405470 (PMC11404154; doi:10.3389/fvets.2024.1405470)

**Supplementary Figures for:**

**Species-level characterization of the core microbiome in healthy dogs using full-length 16S rRNA gene sequencing**

**Figure S1. Microbiome composition is not influenced by sequencing run.** PCoA ordination based on Aitchison distances. Points are color-coded by sequencing run. PERMANOVA analyses support this conclusion (Bray-Curtis F=0.9, R^2^=0.003, p=0.54; Aitchison F=1.35, R^2^=0.004 p=0.09).


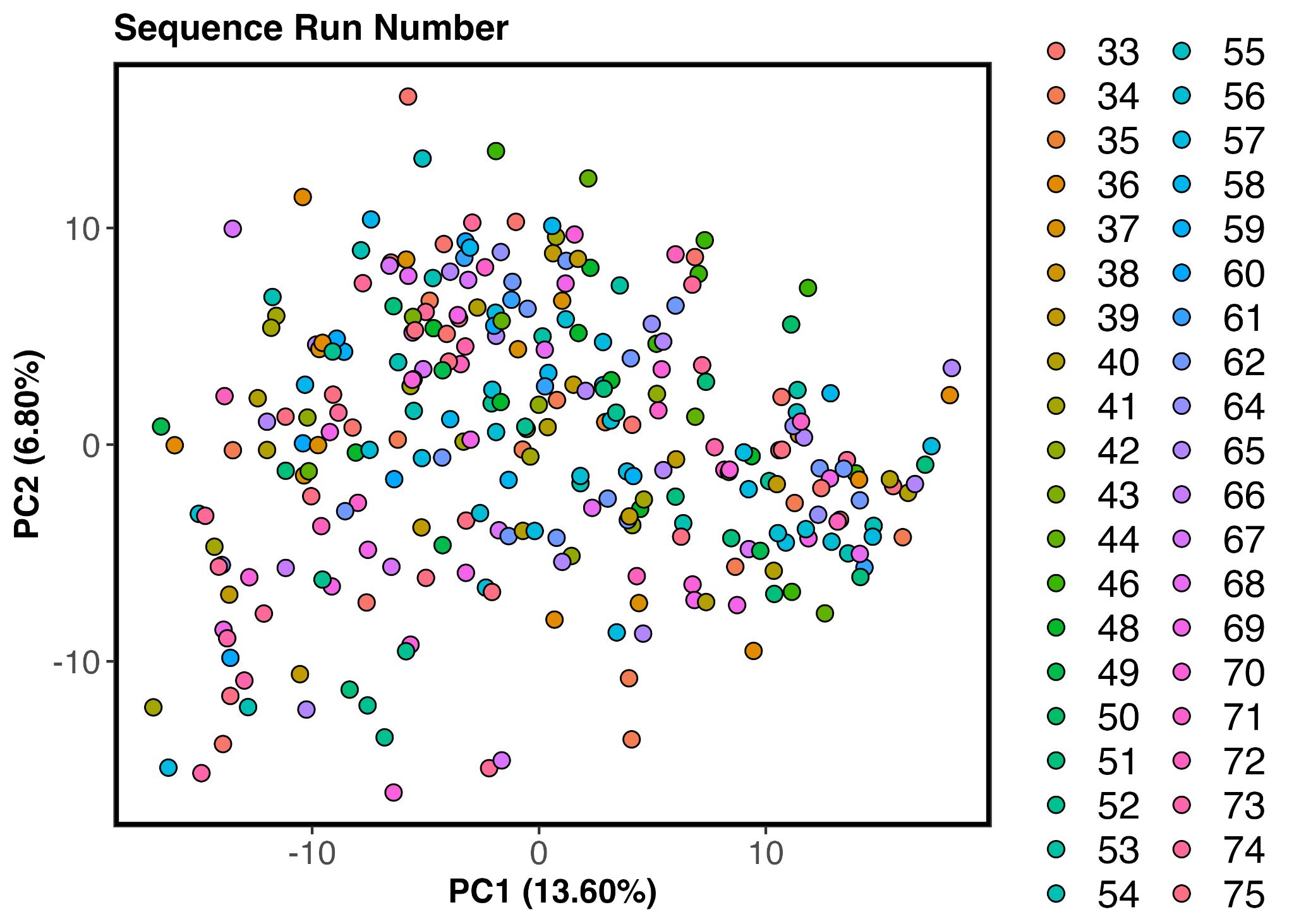


**Figure S2. Microbiomes of dogs who do not have many of the core bacteria.** For these dogs, <40% of their microbiome consisted of core bacteria. They had a different set of microbes present in their fecal microbiomes.


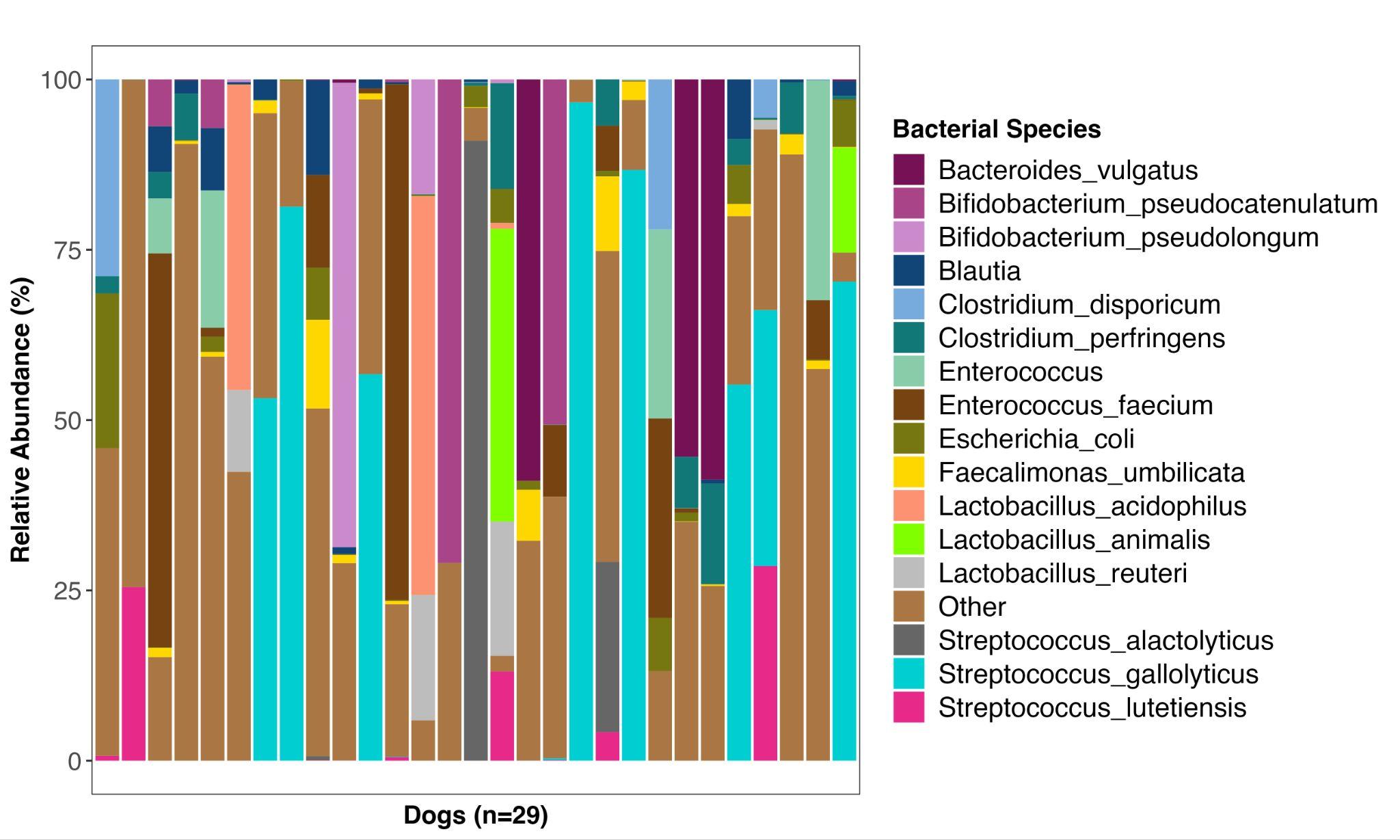

Supplement: Supplementary file 1 [file Data_Sheet_1.docx]
